# Does Weekends Effect Exist in Asia? Analysis of Endovascular Thrombectomy for Acute Ischemic Stroke in A Medical Center

**DOI:** 10.2174/1567202619666220727094020

**Published:** 2022-11-25

**Authors:** Chia-Wei Lin, Hung-Yu Huang, Jeng-Hung Guo, Wei-Laing Chen, Hong-Mo Shih, Hsueh-Ting Chu, Charles C.N. Wang, Tai-Yi Hsu

**Affiliations:** 1 Department of Emergency Medicine, China Medical University Hospital, Taichung, Taiwan;; 2 Doctoral Degree Program in Artificial Intelligence, Asia University, Taichung, Taiwan;; 3 Department of Neurology, China Medical University Hospital, Taichung, Taiwan;; 4 Department of Neurosurgery, China Medical University Hospital, Taichung, Taiwan;; 5 Graduate Institute of Biomedical Sciences, China Medical University, Taichung, Taiwan;; 6 Department of Neuroradiology, China Medical University Hospital, Taichung, Taiwan;; 7 Department of Public Health, China Medical University, Taipei, Taiwan;; 8 Department of Bioinformatics and Medical Engineering, Asia University, Taichung, Taiwan;; 9 Center for Precision Health Research, Asia University, Taichung, Taiwan

**Keywords:** Stroke, working hours, weekends, emergency department, thrombectomy, NIHHS, mRS, diabetes

## Abstract

**Background:**

Discussing the quality measurements based on interrupted time series in ischemic stroke, delays are often attributed to weekends effect. This study compared the metrics and outcomes of emergent endovascular thrombectomy (EST) during working hours versus non-working hours in the emergency department of an Asian medical center.

**Methods:**

A total of 297 patients who underwent EST between January 2015 and December 2018 were retrospectively included, with 52.5% of patients presenting during working hours and 47.5% presenting during nights, weekends, or holidays.

**Results:**

Patients with diabetes were more in non-working hours than in working hours (53.9% vs. 41.0%; *p*=0.026). It took longer during nonworking hours than working hours in door-to -image times (13 min vs. 12 min; *p*=0.04) and door-to-groin puncture times (median: 112 min vs. 104 min; *p*=0.042). Significant statistical differences were not observed between the two groups in neurological outcomes, including successful reperfusion and complications such as intracranial hemorrhage and mortality. However, the change in National Institute of Health Stroke Scale (NIHSS) scores in 24 hours was better in the working-hour group than in the nonworking-hour group (4 *vs*. 2; *p*=0.058).

**Conclusion:**

This study revealed that nonworking-hour effects truly exist in patients who received EST. Although delays in door-to-groin puncture times were noticed during nonworking hours, significant differences in neurological functions and mortality were not observed between working and non-working hours. Nevertheless, methods to improve the process during non-working hours should be explored in the future.

## 1 INTRODUCTION

Guidelines for the early management of patients with acute ischemic stroke have been established [1]. Although endovascular thrombectomy (EST) has become the standard of care for patients with proximal large vessel occlusion, the detailed recommendations for EST are still evolving. Time is still the most critical independent factor affecting the outcomes of patients undergoing EST. Faster revascularization correlates with improved neurological prognosis and smaller infarcts [2]. For every 30-min delay in recanalization, the average absolute rate of optimal outcomes decreases by 11% [3-7]. Previous studies have demonstrated that hospitalization or surgical procedures conducted during nonworking hours may result in worse outcomes than when performed during routine working hours [8-10]. Although this finding is highly debated, the effect was proved after statistical considerations of the severity of the corresponding disease [11]. Many studies have evaluated the effects of workforce quality on surgical procedures conducted in hospitals [12, 13]. This was also discussed under similar circumstances in EST for acute ischemic stroke. However, it has never been discussed in Asian medical institutions. Therefore, this study investigated the procedural times and clinical outcomes in an Asian medical center to explore whether treatment was delayed during nonworking hours.

## 2 MATERIALS AND METHODS

### 2.1 Data Collection

In this retrospective observational study, data of acute ischemic stroke patients with large vessel occlusion who underwent EST between January 2015 and December 2018 in a medical center were analyzed. The study was approved by the hospital’s institutional review board. By protocol, stroke treatment activation occurred when any patient presented to the emergency department and was recognized by the medical staff. We excluded patients who were not activated intially and those who were under 18 years old were excluded from this study. The clinical duty neurologist determined whether to administer thrombolytic drugs, and thrombectomy techniques were performed by an experienced duty neuroradiologist in accordance with the guidelines of the American Heart Association guidelines (AHA). For comparison, the patients were divided into two groups, namely working-hours and nonworking-hours groups. Working hours were defined as 7:00 AM to 7:00 PM on weekdays. Nonworking hours were defined as weekends, official holidays and 7:00 PM-7:00 AM on weekdays. Data extracted for this study included demographics, relevant cardiovascular risk factors, and baseline National Institute of Health Stroke Scale (NIHSS) scores, which were collected by the center’s stroke team.

### 2.2 Outcome Assessment

Time indicators were recorded, including time from symptom onset to the administration of intravenous tissue plasminogen activator (tPA) and presentation to the angiography room. Other time metrics consisted of door-to-image, door-to-angiography room, and door-to-groin puncture times. Outcome data included delta NIHSS, 24 hours (NIHSS score on admission minus NIHSS score 24 hours later), delta NIHSS, discharge (NIHSS score on admission minors NIHSS score on discharge), and the modified Rankin scale (mRS) score at discharge; these were determined through evaluation by neurologists and recorded by specific staff persons. An mRS scores of ≤2 at discharge, and at 30, 90, and 180 days after discharge were considered data reflective of good neurological outcomes. In addition, thrombolysis in cerebral infarction (TICI) score 2b and 3 after thrombectomy were considered good outcomes. Symptomatic and asymptomatic intracerebral hemorrhage as well as mortality were considered data reflective of poor outcomes.

### 2.3 Statistical Analysis

The baseline characteristics of the participants were summarized by using means and standard deviation (SD) for normally distributed continuous variables and by using numbers and percentages for categorical variables. Continuous variables and categorical variables were compared using the two-sample test and the chi-square test, respectively. All statistics were computed using SAS Version 9.4, and a p-value of ≤0.05 was considered statistically significant.

## 3 RESULTS

### 3.1 Demographic Data

Baseline patient demographics are shown in Table **1**. A total of 297 patients receiving mechanical thrombectomy were included in the study, and 58% of them were men, with a median age of 70 years (range:60-78). One hundred and fifty-six (52.5%) ESTs were performed during working hours, and 141 (47.5%) were performed during non-working hours. The median NIHSS score was 18 and 19 for patients presenting to the emergency department in the working and nonworking groups, respectively. Anterior circulation occlusion in 81% and 77% of the working and nonworking group patients, respectively. Significant differences were not observed in the use of intravenous tPA or risk factors, including prior stroke, hypertension, hyperlipidemia, atrial fibrillation, heart failure and cancer in the two groups. Patients without diabetes were overrepresented in the working hours (*p*=0.026).

### 3.2 Time Metrics

The time taken for the procedures is presented in Table **2**. Door-to-image times were longer in the non-working-hour group, with a slightly significant difference (12 min *vs*. 13 min; *p*=0.04). Statistical differences were not observed between the two groups during the time to deliver intravenous tPA or door-to-angiography room. During non-working hours, door-to-groin puncture times were delayed. The median door-to-groin puncture time was 104 min (IQR: 85-131) in the working-hours group and 112 min (IQR:94-140) in the nonworking-hours group (*p =* 0.04). Symptom onset (last known well) to emergency room visit was also slower in patients treated in the nonworking-hours group (274 min vs. 297 min; *p =* 0.049).

### 3.3 Outcomes

Neurological outcomes of the working-hours and nonworking-hours groups are provided in Table **3**. Delta NIHSS, 24 hours was 4 in the working-hours group and 2 in the nonworking-hours group. Statistically significant differences were not observed in the delta NIHSS, 24 hours between the two groups, with a p value of 0.058. In addition, no significant differences were observed in delta NIHSS, discharge, or discharge mRS between the two groups. As for good neurological outcomes, significant differences were not observed in mRS (0-2) after discharge or after 30, 90 and 180 days of discharge between the two groups. Successful revascularization (2b and 3) was achieved in 116 (74%) patients during working hours and in 95 (67%) patients during nonworking hours (*p =* 0.18). Moreover, symptomatic ICH was 7.7% and 12% for the working-hours and nonworking-hours groups, respectively (*p =* 0.2). Despite some differences between the two groups, the mortality rate exhibited no significant differences.

Fig. (**1**) depicts the percentage of mRS(0-2) in each group during different periods. The proportion of nonworking hours was lower than that of working hours in the discharge mRS and 30-day mRS groups. Even though little difference was observed between the discharge mRS and 30-day mRS, the 30-day mRS and 90-day mRS values were significantly different. Furthermore, the neurological prognosis of the two groups improved with time.

## 4 DISCUSSION

Results of this study indicate that the door-to-groin puncture times and door-to-image times were higher in the nonworking-hours group; however, the overall neurological prognosis was similar for both groups. Multiple factors might influence hospital workflow, and some possible causes can be proposed. During nonworking hours, the outpatient clinics do not provide services, leading to crowding in the emergency department on holidays and a slight increase in the door-to-image times. The neurointerventional team in our hospital includes physicians, nurses, and radiologists, some of whom are on-call from home during nonworking hours. Increased door-to-angiography room time was not observed, but significant differences were observed in the door-to-groin puncture time, which may be due to waiting for the anesthesia staff and operators on duty to prepare during nonworking hours. These results, at least in part, are in accordance with those of previous studies that attributed nonworking-hours delays to the availability of nurses, physicians, technicians, and anesthesiologists, all of whom are necessary to commence a procedure after EST code is activated [14, 15]. Previous studies have discussed the “weekends effect”, which means that hospital patients in general [16, 17], emergency department patients [18] and stroke patients presenting during nonworking hours will have a poor prognosis [19-21]. However, many studies have demonstrated that the weekend effect is not relevant to the outcomes of stroke, especially in medical centers, teaching hospitals, stroke centers or current joint commission-certified comprehensive stroke centers [15, 22-24]. The same conclusions have also been noticed in patients who received EST, but mostly in the United States and Europe [25-27]. China Medical University Hospital located in the center of the city, is a medical center with approximately 1,700 admissions of ischemic stroke patients every year. Whenever patients with stroke symptoms or signs visit the emergency department, triage staff or emergency doctors activate the stroke protocol. A neurologist present in the hospital 24/7 decides the management strategy, including whether to administer thrombolytic agent or EST after approaching the patient and analyzing the computerized tomography perfusion (CTP). The CTP report is also evaluated by the neuroradiologist at the same time. After multiple randomized clinical trials provided level 1A evidence for thrombectomy of large vessel occlusions in 2015, the AHA and American Stroke Association (ASA) mandated guidelines for stroke process optimization and time metrics. Taiwan’s National Health Insurance provides comprehensive medical coverage for people. In 2016, Taiwan’s National Health Insurance Administration permitted medical equipment payment for endovascular stroke treatment, leading to an increased number of patients treated by EST in hospitals. Taiwan’s annual average working hours was 2033 in 2018, second only to Singapore in Asia according to international labor statistics published by the Ministry of Labor in 2019. In this study, patients with diabetes were overrepresented in the nonworking-hours group. Job strain may be associated with an increased risk of acute ischemic stroke [28, 29]. Other than the possible mechanisms indirectly caused by job stress, such as unhealthy lifestyle, metabolic syndrome and atherosclerosis, which lead to stroke [30, 31], job strain may directly enhance sympathetic activation and hemostatic or inflammatory conditions [32]. Such patients with acute ischemic stroke but without diabetes present to the emergency department more frequently during working hours than nonworking hours. Diabetes was significantly associated with early neurological deterioration which might lead to poor short-term outcome [33] and this may explain why the proportion of nonworking hours was lower than that of working hours in the discharge mRS (0-2) and 30-day mRS (0-2) groups. Some studies have demonstrated that workforce shortage, decreased nurse-patient and doctor-patient ratios, inexperienced staff, and the patient selection bias for treatment can result in higher mortality [34]. Although the overall mortality and neurological outcomes in this study were not significantly worse during nonworking hours, various aspects, such as door-to-angiography room and door-to-groin puncture time, need improvement. To some degree, this is reflected in the results of delta NIHSS, 24 hours of the two groups (*p =* 0.06). The discharge mRS (0-2) was similar to the 30-day mRS (0-2), with a significant difference between the mRS (0-2) values at 30-day and 90-day periods; these findings are similar to those of other studies [35, 36]. Short-term mRS did not appear to be very accurate in predicting long-term prognosis in this study. Furthermore, the proportion of good functional outcomes increased with time in both the working and nonworking groups after discharge. Given the costs associated with managing hospital administration and the goal of achieving cost-effectiveness, deployment of staff is a critical issue. In a previous study, presentation during nonworking hours was associated with longer door-to-puncture times [37]. Some methods have been proposed to shorten door-to-puncture time by implementing institutional protocols for large vessel occlusion patient triage and treatment [38-40], which might also be helpful in Asia. This study is not without limitations. First, it was a retrospective, single-center design and our small study groups may not be representative of results from other Asian countries. Additionally, there were some changes in eligibility criteria for thrombectomy over the time period according to National Health Insurance payment regulations. Finally, we used one smartphone application to enhance inter-hospital communication and the time delays among referred patients during working hours and non-working hours became unremarkable, especially late in the study.

## CONCLUSION

This study revealed that nonworking-hour effects truly exist in patients who received emergent endovascular thrombectomy in an Asian medical center. Although no significant differences in neurological functions and mortality between the working-hours and nonworking-hours groups after patients with ischemic stroke were treated by EST. However, significant in-hospital delays in periprocedural times were noticed during nights and weekends. These delays may not directly result in an unfavorable outcome, but they should be addressed on a priority basis. Adoption of measures, such as following the AHA and ASA guidelines, establishing a stroke center team, and reviewing the deficiencies in the process regularly, might result in positive outcomes.

## Figures and Tables

**Fig. (1):**
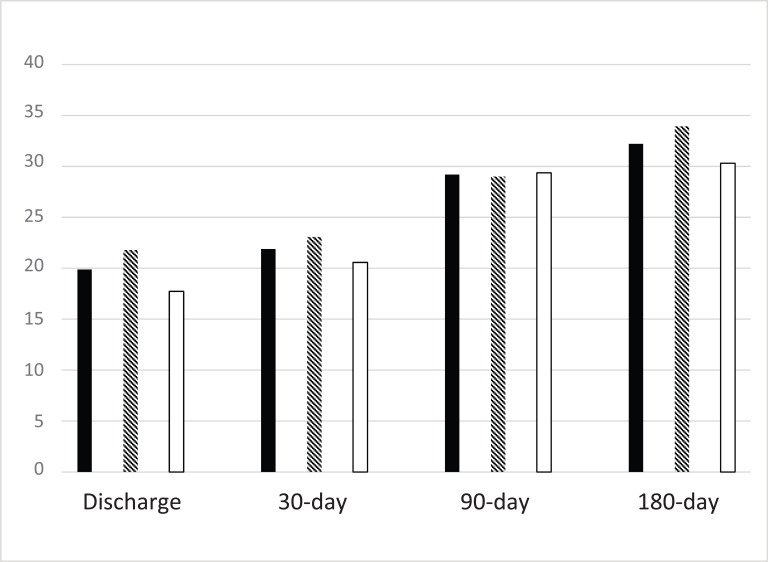
Percentage of mRS(0-2) in each group by different periods.

**Table 1:** Baseline patient demographics.

**Characteristics**	**All (N=297)**	**Working Hour (N=156)**	**Nonworking Hour (N=141)**	***P*-value**
Age, mean ± SD	68.36±12.11	68.88±12.02	67.79±12.22	0.442
Sex, Male, n/N (%)	173(58.25)	98(62.82)	75(53.19)	0.092
Baseline NIHSS, median (IQR)	18.00 (14.00-24.00)	18.00 (14.00-23.00)	19.00 (14.00-24.00)	0.828
Occlusion location	-	-	-	0.381
Anterior circulation	236(79.46)	127(81.41)	109(77.30)	-
Posterior circulation	61(20.54)	29(18.59)	32(22.70)	-
IVT, n/N (%)	97(32.66)	55(35.26)	42(29.79)	0.315
Risk factor	-	-	-	-
Prior stroke n/N (%)	52(17.51)	31(19.87)	21(14.89)	0.259
HTN, n/N (%)	227(76.43)	115(73.72)	112(79.43)	0.246
DM, n/N (%)	140(47.14)	64(41.03)	76(53.90)	0.026
Hyperlipidemia, n/N (%)	55(18.52)	32(20.51)	23(16.31)	0.352
Af, n/N (%)	151(50.84)	82(52.56)	69(48.94)	0.532
HF, n/N (%)	36(12.12)	23(14.74)	13(9.22)	0.145
Cancer, n/N (%)	24(8.08)	15(9.62)	9(6.38)	0.307

**Abbreviations:** SD, Standard Deviation; NIHSS, National Institute of Health Stroke Scale; IQR, Interquartile range; IVT, intravenous thrombolysis; HTN, Hypertension; DM, Diabetes Mellitus; Af, Atrial Fibrillation; HF, Heart Failure.

**Table 2:** Treatment intervals during regular and off-hours.

**Characteristics**	**All (N=297)**	**Working Hour (N=156)**	**Nonworking Hour (N=141)**	***P*-value**
Door to CT perfusion, median (IQR)	12.00 (10.00-17.00)	12.00 (10.00-15.00)	13.00 (10.50-18.00)	0.043
*IVT, median (IQR)	99.00 (82.00-147.0)	102.0 (82.00-129.0)	96.50 (82.00-163.0)	0.320
Door to angioroom, median (IQR)	93.00 (72.00-117.0)	90.00 (69.00-114.0)	98.00 (77.00-124.0)	0.056
Door to groin-puncture, median (IQR)	109.0 (89.00-132.0)	104.0 (85.00-131.0)	112.0 (94.00-139.5)	0.042
Onset to angioroom, median (IQR)	288.0 (206.0-396.0)	274.0 (185.0-388.0)	297.0 (234.0-420.0)	0.049

**Abbreviations:** IVT, Intravenous Thrombolysis; CT, Computed Tomography; IQR, Interquartile Range.

*IVT numbers in each group were showed in Table **1**.

**Table 3:** Outcome characteristics of the patients.

**Characteristics**	**All (N=297)**	**Working Hour (N=156)**	**Nonworking Hour (N=141)**	***P*-value**
*Delta NIHSS, 24hours, mean ± SD	3.10±9.76	4.12±9.85	1.95±9.57	0.058
^#^Delta NIHSS, discharge, mean ± SD	3.34±12.13	3.90±12.57	2.71±11.65	0.398
Discharge mRS, median (IQR)	4.00 (3.00-5.00)	4.00 (3.00-5.00)	4.00 (3.00-5.00)	0.500
Discharge mRS 0-2, n/N (%)	59(19.87)	34(21.79)	25(17.73)	0.380
30-dat mRS 0-2, n/N (%)	65(21.89)	36(23.08)	29(20.57)	0.601
90-day mRS 0-2, n/N (%)	75/257(29.18)	38/131(29.01)	37/126(29.37)	0.949
180-day mRS 0-2, n/N (%)	67/208(32.21)	37/109(33.94)	30/99(30.30)	0.574
TICI2b and 3, n/N (%)	211(71.04)	116(74.36)	95(67.38)	0.185
ICH	-	-	-	-
Symptomatic, n/N (%)	29(9.76)	12(7.69)	17(12.06)	0.205
Asymptomatic, n/N (%)	125(42.09)	71(45.51)	54(38.30)	0.208
Mortality, n/N (%)	35(11.78)	16(10.26)	19(13.48)	0.390

**Abbreviations:** NIHSS, National Institute of Health Stroke Scale; SD, Standard Deviation; IQR, Interquartile Range; mRS, Modified Rankin Scale; TICI, Thrombolysis In Cerebral Infarction; ICH, Intracranial Hemorrhage.

*delta NIHSS, 24 hours: NIHSS score on admission minus NIHSS score 24 hours later,

^#^delta NIHSS score, discharge: NIHSS score on admission minors NIHSS score on discharge.

## Data Availability

The data supporting the findings of the article are available within the article.

## LIST OF ABBREVIATIONS

Af: Atrial Fibrillation
AHA: American Heart Association
ASA: American Stroke Association
CT: Computed Tomography
CTP: Computerized Tomography Perfusion
EST: Endovascular Thrombectomy
HF: Heart Failure
ICH: Intracranial Hemorrhage
mRS: Modified Rankin Scale
NIHSS: National Institute of Health Stroke Scale
TICI: Thrombolysis In Cerebral Infarction
tPA: Tissue Plasminogen Activator
